# Link between cesarean section scar defect and secondary infertility: Case reports and review

**DOI:** 10.5935/1518-0557.20220009

**Published:** 2023

**Authors:** Fathima Mohammed Ahamed, Sadika Solkar, Martina Stevikova, Braulio Peramo Moya

**Affiliations:** 1 Al Ain Fertility Center, Al Jimi area, Al Ain, United Arab Emirates

**Keywords:** cesarean section scar defect, isthmocele, niche, infertility, ultrasound, assisted reproductive techniques

## Abstract

The objective was to study clinical cases and understand the link between cesarean section scar defect with hydrometra and secondary infertility. A retrospective case series from an assisted reproductive center and infertility treatment clinic in the United Arab Emirates. We had five patients with secondary infertility diagnosed with cesarean section scar defect with persistent hydrometra based on high resolution transvaginal ultrasound assessment. The patients underwent surgical repair for the cesarean section scar defect followed by infertility treatment. Transvaginal ultrasound examination showed a normal endometrial cavity with triple lining endometrium and absence of hydrometra; and clinical pregnancy was the main outcome measure. Surgical correction of cesarean section scar defect was successfully performed in the cases presented. The patients had their fertility restored. Clinical studies revealed that cesarean section scar defect may lead to abnormal uterine bleeding, dysmenorrhea, pre-/post-menstrual spotting, heavy or prolonged menses, pelvic pain and secondary infertility. Theoretically, an inflammatory response, such as a wound healing process in the uterus due to hydrometra associated with scar defect may impair embryo implantation. The clinical case studies presented here are based on the correct diagnosis of the cesarean section scar defect with hydrometra and its successful surgical repair. The patients in our study had their symptoms resolved and attained clinical pregnancy.

## Introduction

The number of cesarean sections (C-section) performed is steadily increasing across the world (Boerma *et al*., 2018), despite the 2015 World Health Organization (WHO) statement in favor of vaginal delivery (Betran *et al*., 2016). One of the most common complication of a C-section is a uterine scar with deficient healing, known as an isthmocele, niche or C-section scar defect (Calzolari *et al*., 2019; Sisti *et al*., 2015; Di Spiezio Sardo *et al*., 2017). This condition is defined as a diverticulum on the anterior wall of the uterine isthmus located at a cesarean section scar (Thurmond *et al*., 1999). The prevalence of symptomatic isthmocele after C-section is still unknown with wide variations reported in different studies, ranging between 19.4%-88% (Bij de Vaate *et al*., 2014; Tower & Frishman, 2013).

C-section scar defect can be visualized using transvaginal ultrasound and hysteroscopy (Schepker *et al*., 2015; Fabres *et al*., 2003). A typical transvaginal ultrasound image of a cesarean section scar defect shows a wedge-shaped anechoic area that may partially or totally affect the myometrium (Tulandi & Cohen, 2016; Vikhareva Osser & Valentin, 2010). This finding suggests an impaired healing, although the mechanism is unclear. Impaired healing of the cesarean scar predisposes to the development of a C-section scar impacting pregnancy (Xiao *et al*., 2014). Factors predisposing to poor wound healing include inadequate closure of the uterine incision, postoperative infections, and impaired health conditions such as diabetes or collagen disorders (OuYang *et al*., 2014). In addition, decreased blood flow to the affected tissue predisposes the patient to incomplete or delayed healing (OuYang *et al*., 2014; Ash *et al*., 2007). Roeder *et al*. (2012) evaluated the histopathology of uterine wound healing and found different thicknesses of the myometrium along the scar with disordered muscular fibers and elastosis. Clinically, cesarean section scar defects may cause gynecological complications such as abnormal uterine bleeding (AUB), dysmenorrhea, pre-post-menstrual spotting, heavy or prolonged menses, pelvic pain and secondary infertility (Heller, 2011; Florio *et al*., 2012).

There are only a few studies on the clinical association between secondary infertility and C-section scar defect (Calzolari *et al*., 2019; Gubbini *et al*., 2011; Istvan *et al*., 2017; Vissers *et al*., 2020a; Enderle *et al*., 2020). In fact, the effectiveness of hysteroscopic isthmoplasty in restoring fertility has been demonstrated in only a handful of clinical studies (Gubbini *et al*., 2008; Sanders & Murji, 2018; Tantini *et al*., 2018; Fabres *et al*., 2005). C-section scar defect may contribute to the development of cesarean scar ectopic pregnancy, resulting from embryo implantation within the cesarean section scar tissue (Patel, 2015).

Repair of C-section scar defect is done by using a minimally invasive surgical method such as hysteroscopy or/and laparoscopy and vaginal procedures (Sanders & Murji, 2018; Fabres *et al.,* 2005; Masuda *et al*., 2015; Setubal *et al*., 2018; van der Voet *et al*., 2014; Vervoort *et al*., 2018; Donnez *et al*., 2017; Xie *et al*., 2014; Enderle *et al*., 2020). Other procedures include robotic restoration of the C-section scar defect - but this is limited due to high costs of this procedure, though excellent results have been reported associated with it (Futyma *et al*., 2016; La Rosa *et al*., 2013). Our paper presents five infertile patients diagnosed with cesarean section scar defect with hydrometra and their successful surgical repair to restore fertility.

## Material and Methods

### Study design

*Inclusion criteria*: patients diagnosed with secondary infertility, cesarean section scar defect with persistent hydrometra and no hydrosalpinx (visualized at least thrice in transvaginal ultrasound performed throughout a period of three months); presented to Al Ain Fertility Center between January 2016 and December 2020. *Exclusion criteria:* Infertility cases presented with C-section scar defect without hydrometra.

*Clinical characteristics of patients*: all the patients were aged between 28 to 41 years who underwent surgical management for cesarean section scar defect with hydrometra to treat secondary infertility.

*Diagnosis*: Symptoms related to cesarean section scar defect (chronic pelvic pain, dyspareunia, abnormal uterine bleeding and secondary infertility) were reported. Clinical diagnoses were confirmed by transvaginal ultrasound imaging. *Surgical Treatment:* All the surgical procedures were performed by hysteroscopy and laparoscopy as per explained below: *- Hysteroscopic resection:* The uterine cavity is distended using NaCl solution. Positive pressure is ensured with an automatic pressure infuser. The inferior and superior edges of the defect are resected using a cutting loop and coagulation is performed on the thinnest part of the scar (Calzolari *et al*., 2017; Setubal *et al*., 2018; Donnez *et al*., 2017).

*-Laparoscopic repair:* This surgical technique was described as using a carbon dioxide laser, the scar was opened from one end to the other and fibrotic tissue being excised from the edges of the defect to access healthy myometrium. Before closing, a Hegar probe was inserted into the cervix to preserve continuity of the cervical canal with the uterus. Multiple layers of separate sutures were used to achieve double-layer closure and the peritoneum was then closed (Donnez *et al*., 2017).

*Treatment outcome*: Post-operative transvaginal ultrasound was done to confirm the triple lining normal endometrium and absence of hydrometra. Hormone replacement treatment for endometrial preparation and frozen embryo transfer was done for cases 1, 2, 3 and 5, using the standard protocol (Lawrenz *et al*., 2020; Vissers *et al*., 2020a). Ovulation induction and timed intercourse was the treatment method used for case 4 (Lawrenz *et al*., 2020; Vissers *et al*., 2020a). Pregnancy was confirmed by ultrasound scan in all the cases.

**Patient consent:** This study was a retrospective case series, and Al Ain Fertility Center Institutional Review Board (IRB) approval was obtained before the beginning of the study. The data presented are with complete anonymity of published information and the images used are under non-identifiable category (ultrasound). In addition, careful case-by-case assessment was made to ensure that content is fully anonymous and presents no risk to confidentiality of the study participants.

## Results

**Figure 1:**
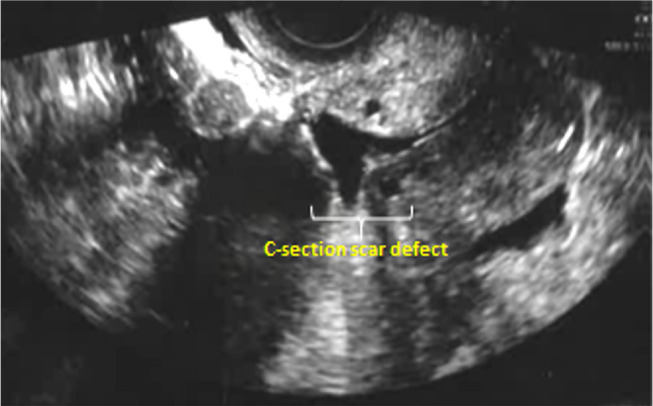
Transvaginal ultrasound examination showing a C- section scar defect.

**Figure 2:**
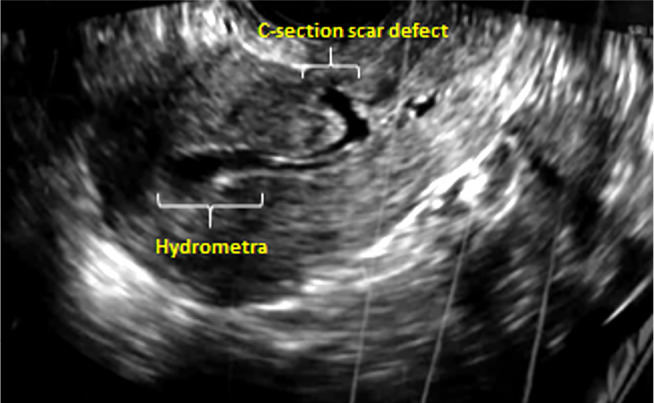
Transvaginal ultrasound examination showing a C- section scar defect, and hydrometra.

**Figure 3:**
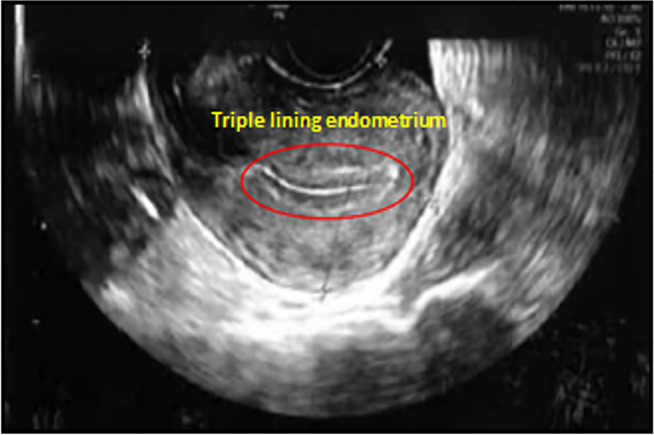
Transvaginal ultrasound examination showing endometrial lining after surgical repair of a caesarean section scar defect (red circle) and the patient had hormone replacement therapy for a frozen embryo transfer.

In a retrospective case series, there were five cases with infertility due to C-section scar defect and persistent hydrometra as per the inclusion criteria from January 2016 and December 2020 presented to our clinic. Clinical characteristics of patients, diagnosis, surgical treatment and outcome measures are summarized in table 1. Patients of age group between 28 to 41 years presented with secondary infertility. High resolution transvaginal ultrasound examination identified a cesarean section scar defect, and hydrometra in all the patients (Figure 1 and 2). In addition, a cesarean section scar defect with hydrometra was confirmed using hysteroscopic assessment. All patients except case 4, underwent *invitro* fertilization (IVF) treatment under standard antagonist protocol with preimplantation genetic testing (Lawrenz *et al*., 2020; Vissers *et al*., 2020a). Blastocysts were biopsied and frozen. Persistent hydrometra was identified in the transvaginal scan at least three times over a period of three months. The patients were then referred for surgery to repair cesarean section scar defect. Hysteroscopic or laparoscopic or combined techniques was performed wherein proximal edges of scar were resected, and repaired (Calzolari *et al*., 2017; Setubal *et al*., 2018; Donnez *et al*., 2017; Enderle *et al*., 2020). After surgery, the patients underwent hormone replacement treatment for endometrial preparation under a standard protocol (Lawrenz *et al*., 2020; Vissers *et al*., 2020a). A triple lining endometrium was visualized in the transvaginal scan and no hydrometra was found (Figure 3). All four patients had euploid embryos transferred. One patient had had a treatment for ovulation induction and timed intercourse (Lawrenz *et al*., 2020). Pregnancy was confirmed by ultrasound assessment. Four out of five patients delivered healthy babies through a cesarean section. Unfortunately, one patient had a miscarriage at the tenth week of pregnancy.

**Table 1:** Summary of the cases presented in the study.

Clinical characteristics of the patients											Diagnosis and treatment				Treatment outcome	
**Case No.**	**Age (year)**	**BMI** **(kg/m^2^)**	**Gravidity**	**Parity**	**Obstetric** **history**	**Previous** **C-sections**	**Duration of** **infertility** **(Year)**	**Infertility factors**	**Hydrosalpinx** **(transvaginal** **ultrasound** **assessment)**	**No. of** **previous IVF** **cycles**	**Ultrasound findings** **(before surgery)**	**Surgical** **treatment** **method**	**Ultrasound** **findings (after** **surgery)**	**ART treatment** **method**	**Pregnancy Test**	**Reproductive outcome**
1	33	35.5	7	4	3 miscarriages, 1ectopic pregnancy	4	2.5	Low ovarian reserve, male factor	Absent	2	C-section scar defect and hydrometra, endometrial polyp	Hysteroscopy and polypectomy	Triple lining endometrium, no hydrometra	^*^HRT for FETStandard protocol- Estradiol and Progesterone	Positive	Uneventful pregnancy. Delivered a healthy baby through C- section
2	41	31	2	2	no relevant history	2	0.83	Tubal factor, advanced maternal age	Absent	1	C-section scar defect and hydrometra	Hysteroscopy guided laparoscopic repair	Triple lining endometrium, no hydrometra	HRT for FETStandard protocol- Estradiol and Progesterone	Positive	Delivered three babies through C- section at 29^th^ week of gestation
3	35	29	5	2	3 miscarriages	2	3	Recurrent miscarriage	Absent	2	C-section scar defect and hydrometra	Hysteroscopy guided laparoscopic repair	Triple lining endometrium, no hydrometra	HRT for FETStandard protocol- Estradiol and Progesterone	Positive	Uneventful pregnancy. Delivered a healthy baby through C- section at 38 weeks of gestation
4	35	28.8	8	3	5 miscarriages, 1 ectopic pregnancy	2	1.8	Recurrent pregnancy loss	Absent	0	C-section scar defect and hydrometra	Hysteroscopy guided laparoscopic repair	Triple lining endometrium, no hydrometra	Ovulation induction and timed intercourse	Positive	Uneventful pregnancy. Delivered a healthy baby through C- section
5	28	29	1	1	no relevant history	1	1.3	endometriosis of ovaries and mild male factor	Absent	1	C-section scar defect and hydrometra	Hysteroscopy guided laparoscopic repair	Triple lining endometrium, no hydrometra	HRT for FETStandard protocol- Estradiol Progesterone	Positive	Miscarriage at tenth week of pregnancy

* Hormone replacement therapy (HRT) for frozen embryo transfer (FET).

## Discussion

The increased number of cesarean sections in recent years can be correlated with an increase in the number of late post-operative complications associated with it (Field & Haloob, 2016). Five clinical cases of secondary infertility studied here presented with a C-section scar defect, which we considered to be the primary cause of infertility, particularly due to the hydrometra associated with the scar defect. Clinical studies revealed that some women who had cesarean sections were not able to conceive due to abnormal uterine bleeding caused by a previous surgical incision (Naji *et al*., 2013; Donnez *et al*., 2017; van den Tweel *et al*., 2019), fluid-filled pouch at the scar site due to impaired wound healing and thinning of the anterior uterine wall (Nezhat *et al.,* 2016). Embryo Implantation is a highly organized process that involves an interaction between a receptive uterus and a competent blastocyst (Thurmond *et al*., 1999; Tower & Frishman, 2013). During implantation, the embryo attaches itself to the endometrial surface of the uterus and any external factor that affects the endometrium may have an influence on this process (Fabres *et al*., 2003; Schugart *et al*., 2008; Vissers *et al*., 2020a; 2020b; Enderle *et al*., 2020). For instance, endometrial polyps, submucosal fibroids or intrauterine device have been listed as factors which may impair implantation (Vissers *et al*., 2020a; 2020b; Enderle *et al*., 2020). From the cases presented, it is suspected that that the inflammatory response or mucus filled hydrometra would negatively impact embryo implantation and may also interfere with sperm motility up to the uterus (Fabres *et al*., 2003; Gubbini *et al*., 2011; Morris *et al*., 1995; Vervoort *et al*., 2015; Vikhareva Osser *et al*., 2009; Schugart *et al*., 2008; Vissers *et al*., 2020a; 2020b; Enderle *et al*., 2020).

The etiology of C-section scar defects may be associated with the number of cesarean sections, labor before cesarean section, uterine position and size of the C-section scar defect (Vikhareva Osser & Valentin, 2010; Wang & Hu, 2015; Shao & Hu., 2015; Vervoort *et al*., 2015; Wang *et al*., 2009; Armstrong *et al*., 2003). Three of the five clinical cases presented here had multiple cesarean section deliveries. Multiple cesarean sections may interfere with tissue perfusion and are also reported to be associated with increased width and depth of the scar defects (Tower & Frishman, 2013; Ofili-Yebovi *et al*., 2008). In addition, surgical interventions such as the level of the uterine incision and the uterine closure technique may cause delayed wound healing (Fabres *et al*., 2005). Maternal obesity and gestational diabetes were also reported to be associated with an increased risk of incomplete wound healing of the uterine incision after previous C-sections (Antila-Långsjö *et al*., 2018).

Clinical consequences of C-section scar defects may include abnormal uterine bleeding (AUB), dysmenorrhea, pelvic pain, postmenstrual spotting, adenomyosis, endometriosis, abscess formation, cesarean scar ectopic pregnancy, and infertility (Tower & Frishman, 2013; Patel *et al*., 2015; Fabres *et al*., 2005; Wang *et al*., 2009; Gonzalez & Tulandi., 2017). A lack of coordinated muscular contractions occurs around the cesarean scar, making the defect collect menstrual debris. Subsequently, the debris leach out through the cervix for several days after menstruation (Thurmond *et al*., 1999). Histopathological studies revealed that congested endometrial fold, lymphocytic infiltration and small polyps in the scar led to abnormal prolonged uterine bleeding (Shao & Hu., 2015). In addition, chronic inflammation and endometrial exfoliation may lead to damaged local blood vessels that cause heavy bleeding (Shao & Hu., 2015). Pelvic pain associated with scar defects could be related to abnormal muscular contraction caused by physiological irregularities in the lower uterine segment (Wang *et al*., 2009). Menstrual blood buildup in the cesarean scar defect due to the presence of fibrotic tissue may reduce uterus contractility around the scar and cause dysmenorrhea (Fabres *et al*., 2003; Morris *et al*., 1995; Sholapurkar, 2018). The secondary clinical consequences may include higher risk of complications during gynecological procedures such as uterine evacuation, hysterectomy, endometrial ablation, and insertion of an intrauterine device (Betsy *et al*., 2012).

The frequency of scar defects increases with the increase in the number of cesarean sections due to decrease in residual myometrium thickness (Fonda *et al*., 2011). Scars with defects are located lower in the uterus than intact scars (Vikhareva Osser *et al*., 2009). These gynecological disorders may cause secondary infertility. Another important clinical consequence of cesarean section scar defect is cesarean scar ectopic pregnancy and uterine rupture in a subsequent pregnancy (Tower & Frishman, 2013). Although the mechanism is unclear, it is proposed that impaired wound healing of the previous cesarean section scar predisposes to the development of a scar-impaired pregnancy (Xiao *et al*., 2014; Gonzalez & Tulandi., 2017). Clinical case 4 presented in our study reported scar-impaired pregnancy which yielded a miscarried. A clinical study reported that gestational sac implanted over a large cesarean section scar defect led to spontaneous miscarriage (Szkodziak *et al*., 2019). In the clinical case no. 5, miscarriage occurred at the tenth week of pregnancy, even though we couldn’t associate it with a scar defect. A C-section scar defect diagnosis can be clinically suspected from a history of cesarean sections and typical clinical symptoms, such as abnormal uterine bleeding, dysmenorrhea and infertility (Heller *et al*., 2011; Florio *et al*., 2012; Chen *et al*., 2014). Although, there is no established standard diagnostic criteria for C-section scar defects (Tower & Frishman, 2013; Kremer *et al*., 2019), it can be confirmed by using transvaginal ultrasonography and diagnostic hysteroscopy (Schepker *et al*., 2015). Normal endometrial cavity with a proper endometrial lining is one of the most important factors which need to be assessed during infertility evaluation and treatment (Thurmond *et al*., 1999; Bij de Vaate *et al*., 2014; Tower & Frishman, 2013; Schepker *et al*., 2015; Fabres *et al*., 2003, Vissers *et al*., 2020a; 2020b; Enderle *et al*., 2020). In the cases presented hereby, a C-section scar defect with hydrometra was visualized at least thrice in transvaginal ultrasound performed along a period of three months. The presence of fluid-filled endometrial cavity depicts an abnormal uterine anatomy and absence of hydrosalpinx. Surgical techniques to repair scar defects include laparoscopic surgery, hysteroscopic surgery (resectoscopic treatment), laparoscopic surgical repair with hysteroscopic assistance and vaginal procedure (Calzolari *et al*., 2019; Tower & Frishman, 2013; Setubal *et al*., 2018; van der Voet *et al*., 2014). Endoscopic treatment is a commonly used method for the correction of a C-section scar defect (Tantini *et al*., 2018). In hysteroscopic surgery, the lower and upper edges of the defect are resected using a cutting loop and the thinnest part of the scar is coagulated (Vervoort *et al*., 2018). Whereas, in laparoscopic surgery, the scar is completely resected and sutured using a combination of laparoscopy and hysteroscopy (Tanimura *et al*., 2015). C-section scar defect repairs using minimally invasive approaches are reported to be efficient in achieving reduced clinical symptoms and restore secondary infertility (Gubbini *et al*., 2011; van der Voet *et al*., 2014). López Rivero *et al*. (2019) reported a clinical case of secondary infertility, due to a scar defect with persistent hydrometra that was hysteroscopically corrected to restore fertility. Istvan *et al*. (2017) demonstrated that 80% of the patients diagnosed with a C-section scar defect that had surgical treatments (hysteroscopic and laparoscopic isthmoplasty) became pregnant within 24 months and delivered before 36 months of treatment. In cases of infertility treatment, the reproductive performance after the scar defect correction surgery shows the effectiveness of the accurate diagnosis and treatment of patients using efficient techniques (Istvan *et al*., 2017). In our study, all the patients underwent surgical repair of the scar defect with hydrometra, and fertility was restored. All the clinical cases presented in our study showed scar defects with hydrometra. Surgical correction of the scar defect resolved the hydrometra, and the patients were able to get pregnant by IVF treatment.

Hysteroscopic surgery may not be performed in patients with a myometrial thickness of less than 2 mm surrounding the scar defect, and defects cannot be sutured hysteroscopically (Xie *et al*., 2014). Whereas vaginal surgery has no minimum requirement for myometrial thickness because uterine perforation is not a concern. In addition, they reported that even though vaginal surgery has a longer operating time and greater blood loss, it has a higher therapeutical efficacy rate compared to operative hysteroscopy (Xie *et al*., 2014). Even though hysteroscopic and laparoscopic corrections are highly effective in the treatment of cesarean section scar defects, the chances of recurrence and further complication of the condition cannot be completely eliminated (van der Voet *et al*., 2014; Kremer *et al*., 2019; Shao & Hu., 2015). Thus, there is a need for an extensive investigation and analysis of the techniques used for the treatment of scar defects. The decision to treat and treatment method may be chosen by considering the severity of the condition, characteristics of the scar defect, and patient’s desire for future pregnancy (Baranowski *et al*., 2020). A clinical study highlights the need for an increased awareness towards the potentially adverse impact of a scar defect on ART treatment (Lawrenz *et al*., 2020). There is a risk of developing intra-cavitary fluid during ovarian stimulation in patients with scar defects that may cause an increase in the circumference of the scar defect and increase difficulties during embryo transfer (Lawrenz *et al*., 2020). It is important that the scar defect corrective surgery needs to be performed by a skilled and experienced surgeon. Clinical studies on understanding precise clinical symptoms, proper diagnosis, and efficiency of different treatment approaches used in the management of gynecological complications and secondary infertility associated with cesarean section scar defects is vital in planning and providing appropriate medical services.

## Conclusion

Cesarean section scar defects can be a reason for infertility, especially when the endometrial cavity is filled up with fluid (hydrometra), and when no normal endometrial lining can be visualized. Studies postulated various mechanisms by which scar defect hydrometra may interfere with embryo implantation (Gubbini *et al*., 2011; Setubal *et al*., 2018; Vervoort *et al*., 2018; Vissers *et al*., 2020a). Laparoscopic or hysteroscopic approaches are used in the surgical treatment of cesarean section scar defects. Making an accurate diagnosis is critical for cases with a C-section scar defect for timely treatment and reversal of the condition. Not all scar defects may cause symptoms or subfertility that requires more clinical and follow up studies. It is to be noted that at present, there is no conclusive evidence from clinical studies about the efficiency of the surgical procedure in the management of C-section scar defects and restoring fertility. Our study demonstrated that C-section scar defects can be repaired surgically thus restoring normal anatomy and preventing fluid to reach the endometrial cavity. As a result, a normal endometrial lining can be visualized and the patients attained pregnancy afterwards with assisted reproduction techniques, such as ovulation induction and timed intercourse, or IVF treatment. The association between a C-section scar defect and fertility should be subjected to future studies for better management.
